# Weakened continental lithosphere beneath the northern Red Sea inferred from elastic thickness

**DOI:** 10.1038/s41598-024-64801-2

**Published:** 2024-06-14

**Authors:** Chokri Jallouli, Waleed Al-Dini, Saad Mogren, Hassan Alzahrani

**Affiliations:** Department of Geology and Geophysics, College of Science, King Saud University, Riyadh, 11451 Kingdom of Saudi Arabia

**Keywords:** Northern Red Sea, Lithosphere, Flexural rigidity, Elastic thickness, Gravity, Bathymetry, Planetary science, Solid Earth sciences

## Abstract

The northern Red Sea (NRS) is considered an extended continental region that has resulted in a rift system. Gravity and bathymetry data were used to estimate the Moho depth and the elastic thickness ***Te*** of the lithosphere beneath the NRS region to characterize its flexural rigidity and understand its mechanical behavior. Focusing on the Mabahiss Deep in NRS, we analyzed the lithosphere's flexural rigidity. The observed long-wavelength positive Bouguer anomaly is attributed to crustal thinning and lithospheric mantle uplift. The crustal thickness varies from 28 km in coastal areas to 24 km beneath the axial rift, supporting a regional compensation model over the Airy model. Forward modeling suggests that the optimal model explaining the regional Bouguer anomaly is a flexural model with ***Te*** equal to 7 km, indicating a weak and irregular continental crust. The primary factor contributing to this weakness is heating activity. Given the weakened state of the crust and the ongoing extension in the region, the NRS rift could evolve into a rupture, potentially leading to the formation of oceanic crust.

## Introduction

The development of the Red Sea is attributed to an active divergence boundary between the Arabian Shield and the Nubian (African) Shield. This boundary has resulted in a narrow ocean width of approximately 300 km. The Red Sea shows a narrow, deep-water axial zone at its center, flanked by marginal shelves and coastal plains showing a series of Cenozoic sediment layers underlain by a faulted basement. The northern Red Sea (NRS) is particularly notable for its high seismic activity, especially along the axial depression^1,2^. Using the S-wave receiver function, there is evidence of a considerable lithosphere thinning from the Arabian Shield in the west toward the axial trough of the Red Sea^3^. The lithosphere-asthenosphere boundary varies from 160 km in the eastern Arabian shield to about 60 km in the coastal area, with a trend to become much thinner towards the axial trough of the Red Sea^3^. Such a considerable thinning of the lithosphere is the result of divergence between the Arabian plate and the Nubian plate.

**Figure 1:**
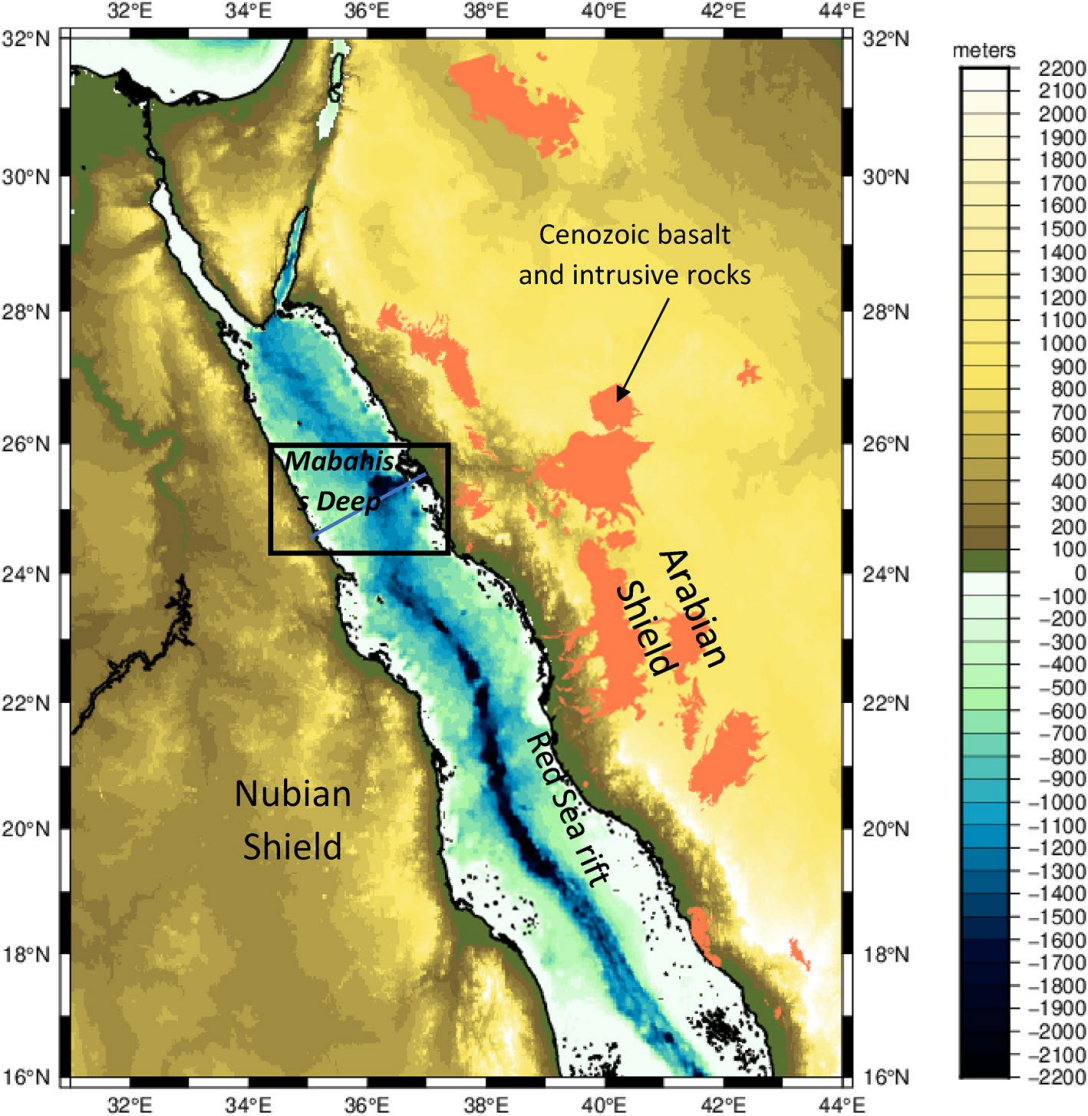
Topographic map of the Red sea, Arabian Shield and Nubian Shield extracted from ETOPO1^18^. The black square represents the study area's location including a depression zone shown in Fig. 3: the Mabahiss Deep. The blue line represents the profile’s location shown in Fig. 6. The map is generated using GMT6.0 software (https://www.generic-mapping-tools.org).

Various geophysical studies have been conducted on the Red Sea crust^1,2,4–10^. These studies reveal that the Red Sea is a rift system displaying different stages of ocean development, from the continental rifting stage to the lithosphere drifting and ocean spreading. An active seafloor spreading is occurring in the southern section of the Red Sea region, a transition zone in the center and late stages of continental rifting in the North (Fig. 1). Therefore, the mechanical behavior of the lithosphere beneath the Red Sea region differs from South to North.

The elastic thickness of the lithosphere is a crucial parameter for understanding its mechanical behavior and its strength. It plays a significant role in various geological processes, such as plate tectonics and crustal deformation. Determining the elastic thickness provides insights into the flexural rigidity and the ability of the lithosphere to support loads. This study aims to understand the mechanical behavior of the lithosphere beneath the northern Red Sea region, characterized by a stretched continental lithosphere. We imaged the shape of the Moho discontinuity and estimated the elastic thickness (***Te***) using gravity and bathymetry data. The elastic thickness can be interpreted in terms of thermal and mechanical structures of the lithosphere. In general, low values of ***Te*** referring to a weak lithosphere, are observed in active tectonic areas and rifts^11^. The elastic thickness of the lithosphere can be estimated through the flexural response of the lithosphere to loads. This approach relies on the principle of isostasy, which considers the balance between the vertical forces acting on the lithosphere and the underlying asthenosphere. The flexural response of the lithosphere to surface loads depends on the rigidity of the lithosphere. Indeed, the rigidity is among factors that control the vertical displacement of the plate. Measuring rigidity is very important for understanding the mechanical behavior of the lithosphere. There is a strong dependence of the rigidity of the lithosphere (***D***) on its elastic thickness (***Te***). Various methods are used to estimate ***Te*** including spectral techniques such as admittance or coherence between gravity and bathymetric data^12^, continuous wavelet transform^13–15^, and forward modeling of the gravity data^11^. However, methods based on spectral analysis may be biased by several factors and often provide an inconsistent estimate of ***Te,*** especially if the size of the study area is small^12,16^. In this work, we employed a forward modeling approach to estimate ***Te*** of the lithosphere beneath the northern Red Sea. By comparing observed gravity data with predicted values, we determined the appropriate elastic thickness that best fits the observed flexural response.

## Gravity and bathymetry data analysis

**Figure 2:**
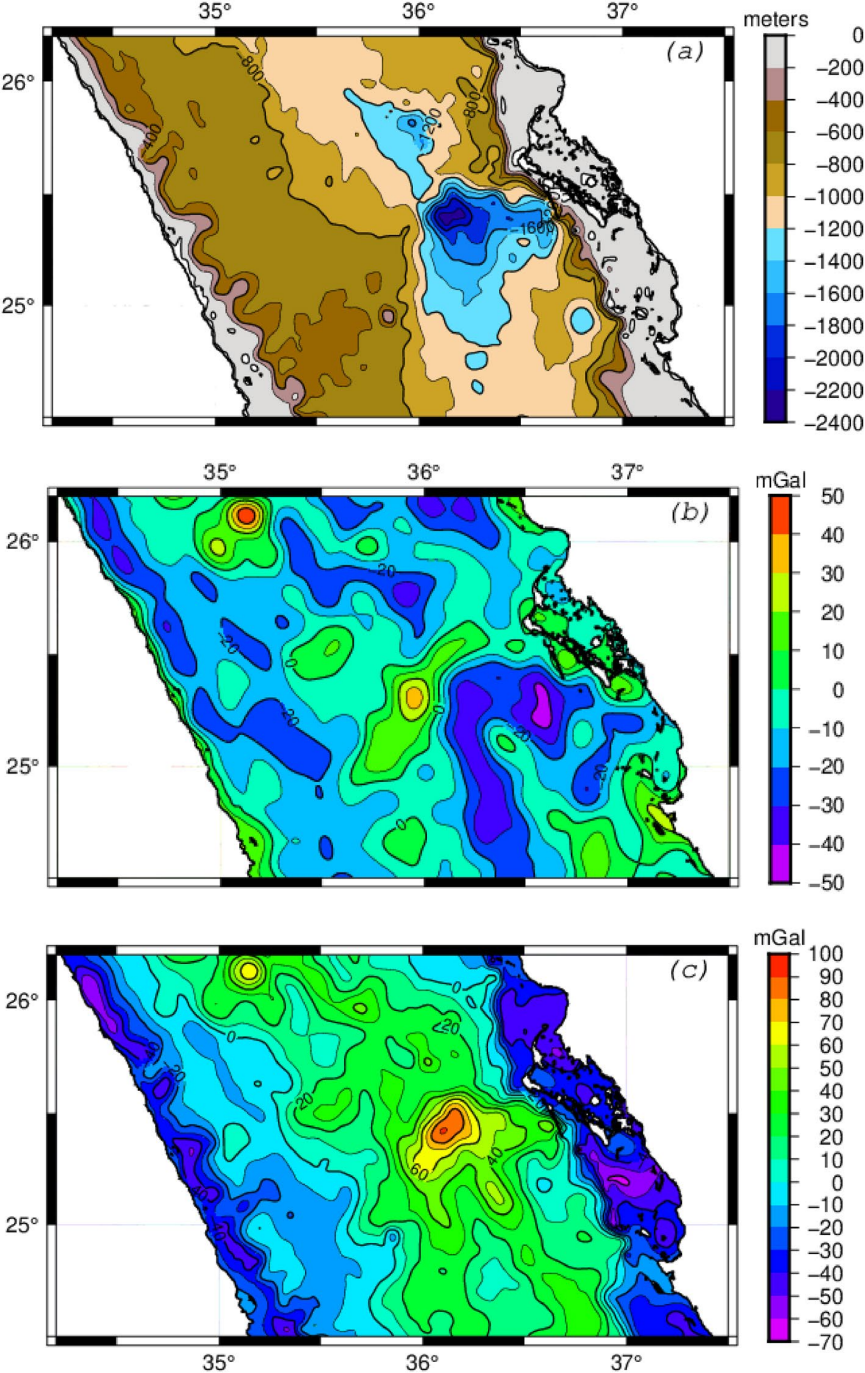
**(a)** Bathymetry map of the NRS region extracted from ETOPO1^18^. The depression in the center of the map is known as Mabahiss Deep where the depth exceeds 2200m. **(b)** Free-air anomaly map extracted from WGM2012^17^. **(c)** Bouguer anomaly map for the NRS region obtained by correcting for the water layer effect using densities of 2.67 g/cm^3^ for the crust and 1.035 g/cm^3^ for the seawater. The Bouguer anomaly reaches 100 mGal over Mabahiss depression. The observed Bouguer anomaly decreases and becomes negative toward the coastal zones. All maps are generated using GMT6.0 software (https://www.generic-mapping-tools.org).

**Figure 3:**
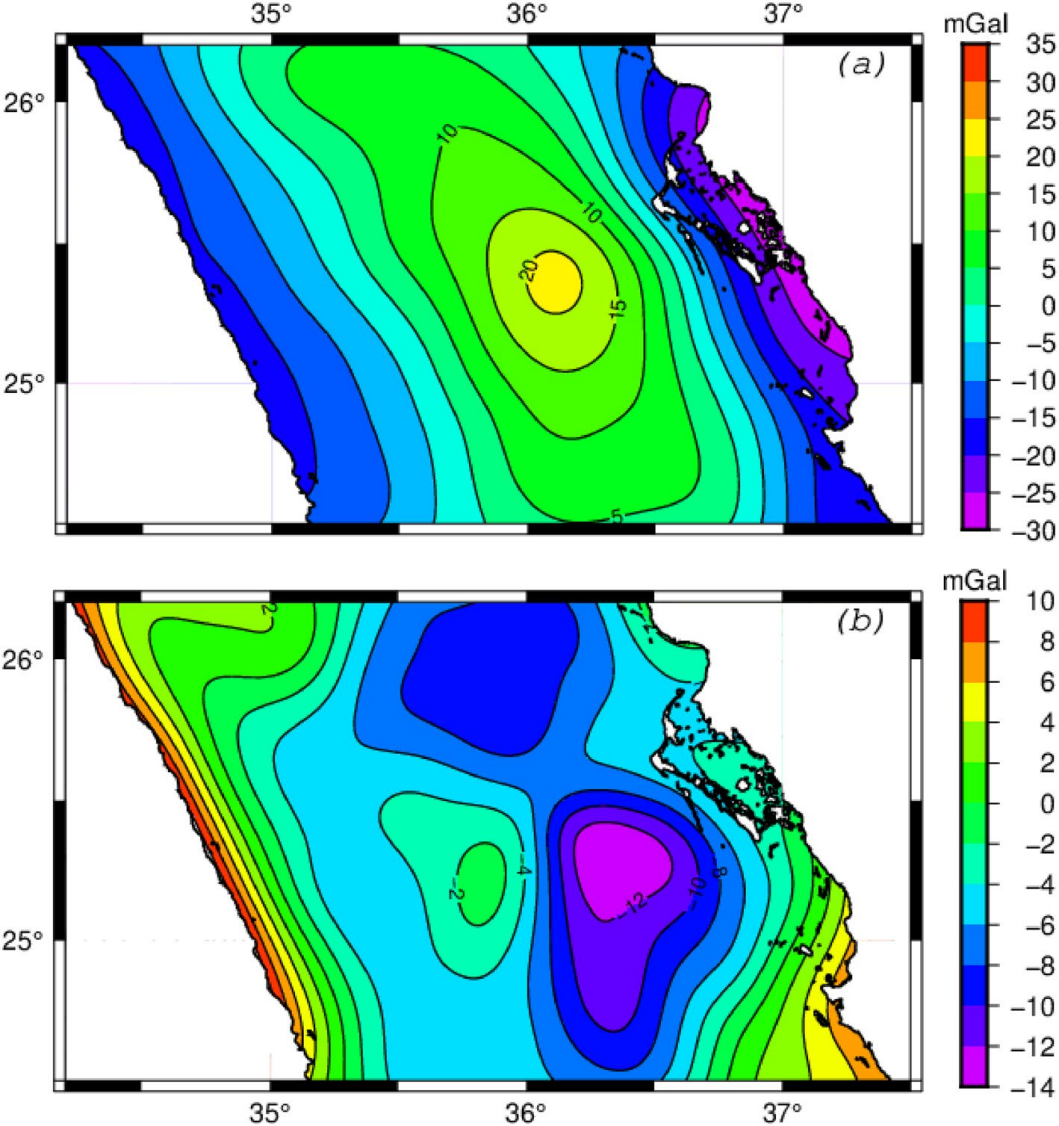
**(a)** Map showing the regional Bouguer anomaly obtained by upward continuation to 25km. Contour interval: 20 mGal. All short-wavelength anomalies caused by the heterogeneities within the uppermost crust are attenuated. The map shows a long-wavelength anomaly trending along the Red Sea and increasing toward the sea axial rift. This anomaly is caused mainly by the Moho discontinuity. **(b)** Isostatic anomaly obtained by extracting the gravity effect of the Moho discontinuity assuming Airy model (Fig. 5a) from the upward Bouguer anomaly (**a**) using density contrast across Moho discontinuity 0.35 g/cm^3^. Maps are generated using GMT6.0 software (https://www.generic-mapping-tools.org).

The vertical displacement of the lithosphere is controlled by its rigidity. The rigidity, or elastic thickness, of the lithosphere can be assessed through an analysis of gravity and bathymetry data. Examination of the gravitational field enables the identification of regions with positive or negative anomalies caused by lateral density variations below the surface that may be induced by the thickness variation of the crust. We used the Free-air gravity anomalies (Fig. 2b) extracted from the WGM2012 model^17^. These global anomalies prove highly valuable in revealing regional and deep crustal structures, as manifested by long-wavelength anomalies. As the free-air anomalies include the effect of the deficiency of mass caused by seawater, we calculated Bouguer anomalies using bathymetry data derived from the Global relief model ETOPO^18^. The bathymetric map of the selected area shows the Mabahiss deep zone in NRS (Fig. 2a), where the seawater depth exceeds 2200m. Bouguer anomalies (Fig. 2c) were derived by eliminating the influence of mass deficiency caused by seawater from the observed free-air anomalies. This was achieved using average density values of 2.67 and 1.035 g/cm^3^ for the crust and water, respectively. The resulting Bouguer anomalies exhibit both long- and short-wavelengths attributed to density variations in the subsurface, including the real root of the crust. The short-wavelength anomalies are associated with shallow-depth heterogeneities rather than regional lithospheric structure heterogeneities. To better observe the gravity response of the crustal root, which is expressed by long-wavelength anomalies, we attenuated short-wavelength anomalies using an upward continuation process^19^. Multiple upward continuations were calculated for different elevations until all short-wavelength anomalies caused by shallow heterogeneities within the crust were attenuated, achieving this at an upward distance of 25 km (Fig. 3a). The upward Bouguer anomaly map reveals a positive gravity trend across the axial trough of the Red Sea, then it decreases and becomes negative toward coastal zones. This observed long-wavelength anomaly is a result of crustal thinning along the Red Sea trough, expressed by an anti-root of the Moho discontinuity. This anti-root is the response of the lithosphere to compensate for the overall mass deficiency beneath sea level. This structure of the crust infers a weakness of the lithosphere in the NRS. The observed free-air anomalies (Fig. 2b) show that the deficiency of mass represented by the sea water is not fully compensated. Indeed, in the case of full compensation, the free-air anomalies should be close to zero. However, in the study area, we observe both negative and positive free-air anomalies with some correlation to the bathymetry. The degree of compensation expressed by the anti-root depends on the flexural rigidity of the lithosphere, which is strongly related to its elastic thickness (***Te***).

## Modeling of the elastic thickness (***T***_***e***_)

The flexural response of the lithosphere to surface loads is primarily determined by its rigidity, which controls the vertical displacement of the plate. There is a strong dependence of the rigidity of the lithosphere (***D***) on its elastic thickness (***Te***). Based on the flexure theory employed to simulate the Earth's lithospheric behavior under surface loads, the flexural rigidity ***D*** of the lithosphere is expressed as follows^35^:

$${\varvec{D}}= \frac{E{{\varvec{T}}{\varvec{e}}}^{3}}{12(1-{\sigma }^{2})}$$

with ***E*** is the Young’s modulus of the lithosphere, ***σ*** is the Poisson’s ratio of the lithosphere.

To assess the elastic thickness, scientists employ theoretical models and numerical techniques grounded in the flexural behavior of an elastic plate. By juxtaposing observed gravity against predicted values, we can ascertain the optimal elastic thickness that most accurately corresponds to the flexural reaction of the studied lithosphere.

**Figure 4:**
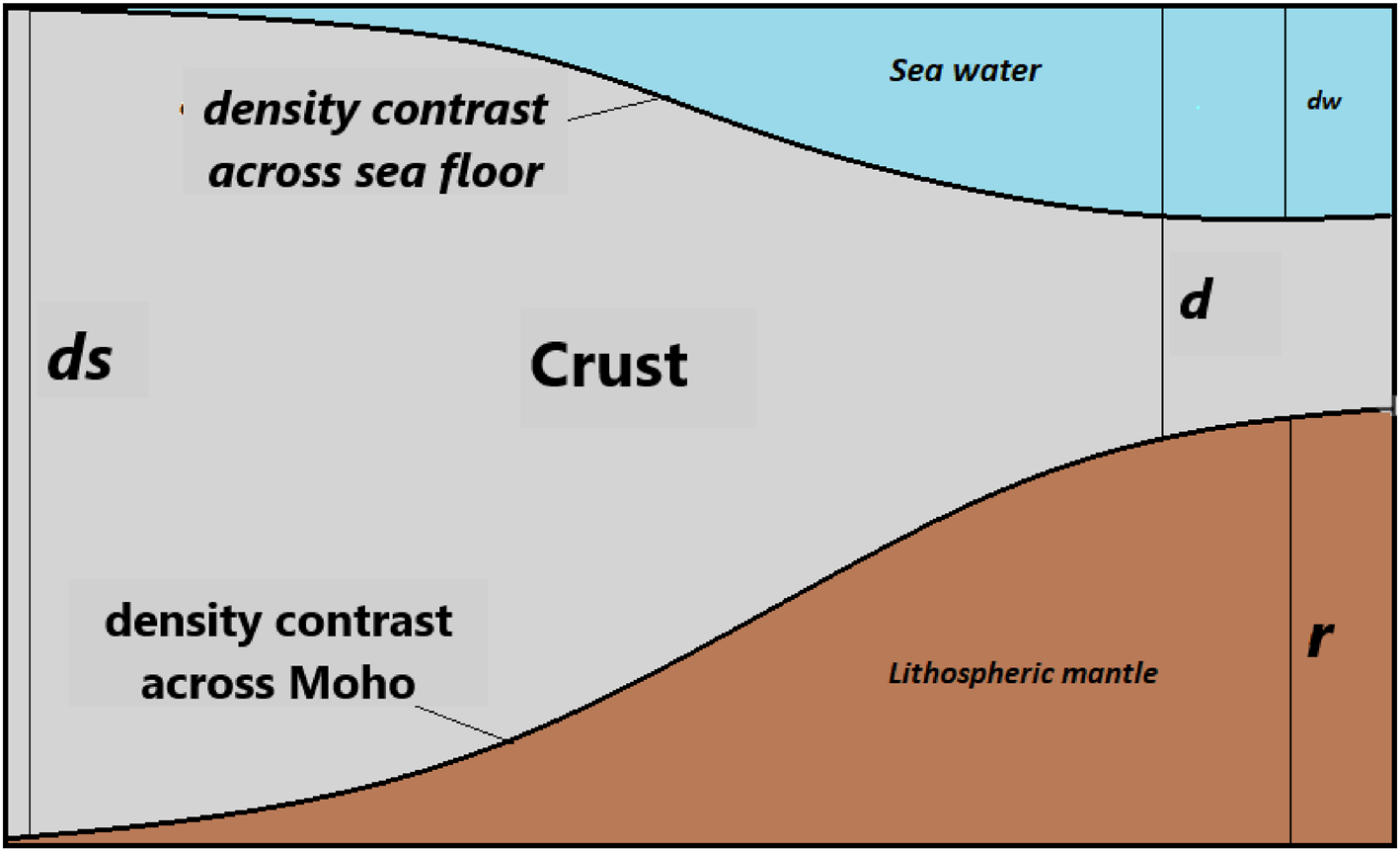
Geometry of compensation of masses in the local compensation model (Airy model). ***d***_***w***_ depth of the seafloor; ***d***_***s***_ normal thickness of the crust in the coastal zone; ***r*** anti-root of the crust; ***d*** depth to Moho discontinuity; ***ρ***_***t***_ density of the uppermost of the crust; ***ρ***_***w***_ sea water density; ***ρ***_***c***_ lower crust density; ***ρ***_***m***_ the uppermost mantle density; ***Δρ***_***w***_ density contrast across seafloor; ***Δρ***_***m***_ density contrast across Moho discontinuity.

In the first stage, we evaluated the effectiveness of the Airy model. This model assumes that the Earth's crust is buoyant on a denser, more viscous layer. It offers a simplified calculation of the Moho discontinuity depth by assuming that the mass deficit, represented by seawater, is entirely compensated by a rise or an uplift of the lithospheric mantle, resulting in an excess mass beneath the crust (Fig. 4). In this case, the depth of the Moho discontinuity can be estimated using the following equation^20^:

$${\varvec{d}}= {d}_{s}- \left(\frac{{{\varvec{\rho}}}_{t}-{{\varvec{\rho}}}_{w}}{{{\varvec{\rho}}}_{m}-{{\varvec{\rho}}}_{c}}\right){d}_{w}$$

$${\varvec{d}}= {d}_{s}- \left(\frac{{\boldsymbol{\Delta }{\varvec{\rho}}}_{w}}{{\boldsymbol{\Delta }{\varvec{\rho}}}_{m}}\right){d}_{w}$$

$${\varvec{d}}\boldsymbol{ }\approx {d}_{s}-4.67 {d}_{w}$$

where ${{\varvec{\rho}}}_{m}$ and ${{\varvec{\rho}}}_{c}$ are the density of the uppermost mantle and the lower crust. ${{\varvec{\rho}}}_{t}\text{ and }{{\varvec{\rho}}}_{w}$ are the density of the uppermost crust and seawater. ${{\varvec{d}}}_{{\varvec{s}}}$ is the normal thickness of the crust. ${{\varvec{d}}}_{{\varvec{w}}}$ is the depth of the sea bottom.

**Figure 5:**
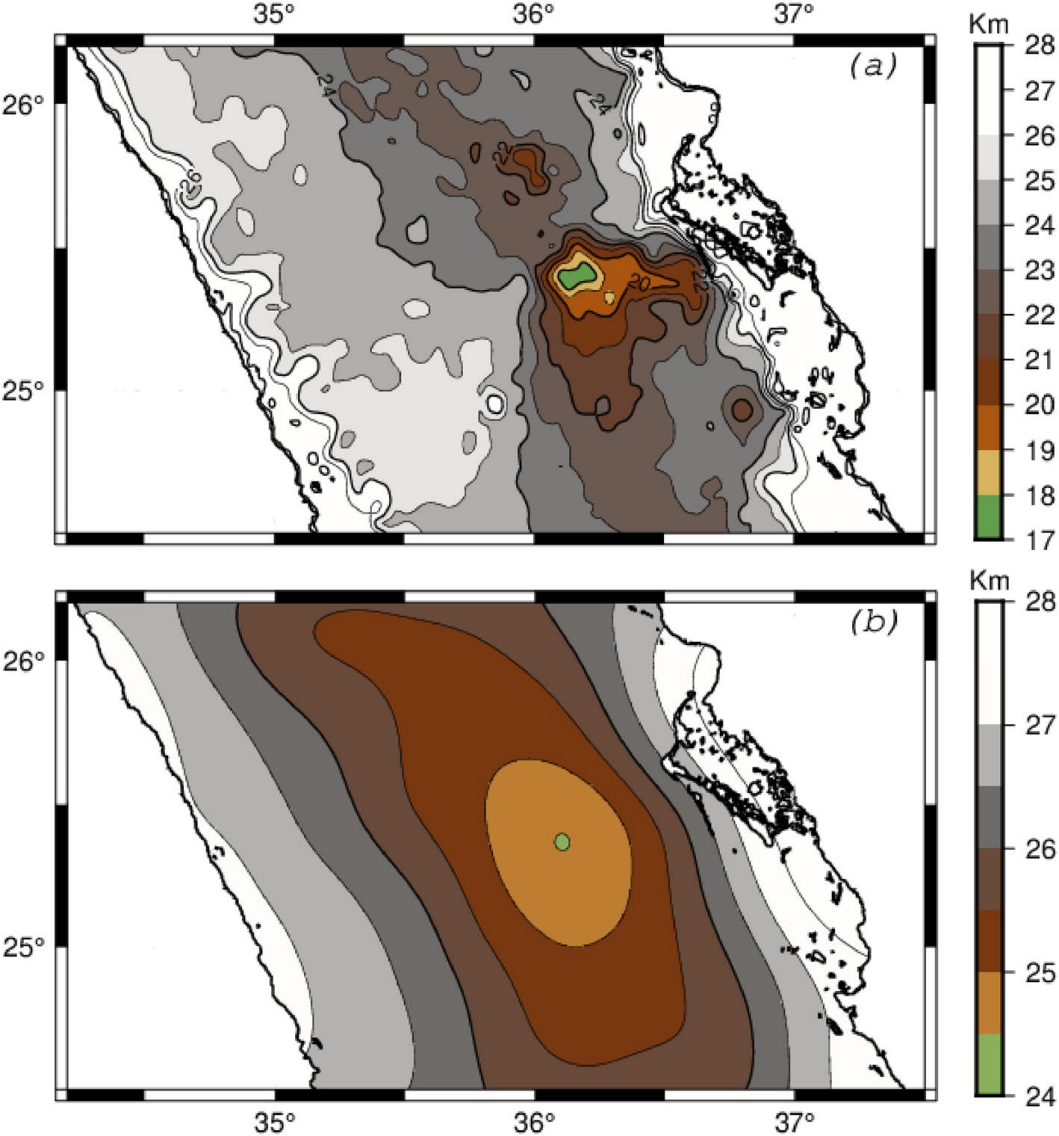
**(a)** Map showing the depth of the Moho discontinuity assuming the Airy model, **(b)** Depth of the Moho derived from the regional gravity anomaly (Fig. 3a). Maps are generated using GMT6.0 software (https://www.generic-mapping-tools.org).

For the NRS lithosphere, based on the seismological study^3^, ${{\varvec{d}}}_{{\varvec{s}}}$ is estimated to be 28km, the density of the uppermost mantle (${{\varvec{\rho}}}_{m}$) is 3.2 g/cm^3^ and the density of the lower crust (${{\varvec{\rho}}}_{c}$) is 2.85 g/cm^3^. For the density of the uppermost crust and sea water, we used the standard values, 2.67 g/cm^3^ and 1.035g/cm^3^. Using the formula above, we calculated the Moho depth. Figure 5a shows the predicted Moho depth map of the NRS assuming the Airy model. It shows that the depth varies from 28 km in the coastal area, then decreases toward the axis of the Red Sea, where it reaches 18 km. The fully compensated Airy model suggests an anti-root in NRS trough of approximately 10 km. To verify the reliability of this crustal thinning amount, we calculated the gravity anomalies resulting from this anti-root using Parker's method^21^. By considering the misfit between the observed anomaly and the calculated anomalies based on the Airy model, namely the isostatic anomaly that reaches −13 mGal (Fig. 3b), it’s clear that the NRS lithosphere thinning could not be explained by the Airy isostatic model which it assumes a full compensation. A regional-type isostatic mechanism assuming a flexural model is more suitable to explain the observed Bouguer anomaly than a local mechanism. Additionally, assuming that the long-wavelength gravity anomaly obtained through upward continuation (Fig. 3a) is attributable to the Moho discontinuity, we deduced the Moho depth (Fig. 5b). The resulting map illustrates a decrease in the depth of the Moho discontinuity from 28 km in coastal areas to 24 km in the axial rift. The thinning of the crust from coastal areas to the Red Sea axial trough is less than 4 km, whereas it’s estimated to be 10 km using the local isostatic model. This finding confirms that the lithosphere has a certain rigidity, suggesting that the flexural model is better suited to compensate for the mass deficiency of Mabahiss Deep.

A question arises: how much is the rigidity of the NRS lithosphere? To measure the flexural rigidity, we estimated the elastic thickness using the iteration process. A forward modeling using Parker’s method^21^ is used to calculate the gravity anomalies of a surface or interface. This method is widely used in potential field data processing and interpretation^22^. It’s a spectral method to calculate the potential anomaly. To achieve this, the interface function is transformed into Fourier series using the FFT algorithm. The method has proven to be very useful in forward problems^23^. In the present study, we used the GRAVFFT-GMT module^24^. This software computes the gravity response due to flexure induced by a surface load represented by the topography. In our case, this load represents a deficiency of mass caused by seawater presence which can be visualized through bathymetry data. The software takes the 2-D forward FFT of the grid and uses the full Parker’s method applied to the chosen elastic plate characterized by a given elastic thickness ***Te***. We applied this algorithm to calculate gravity anomalies using different regional isostatic models with different values of ***Te***, then we compared the subsequent predicted gravity anomaly with the observed Bouguer anomaly. We tested different values of ***Te***, from 1 to 12 km, with an increment of 1 km, and we calculated the RMS for each curve.

**Figure 6:**
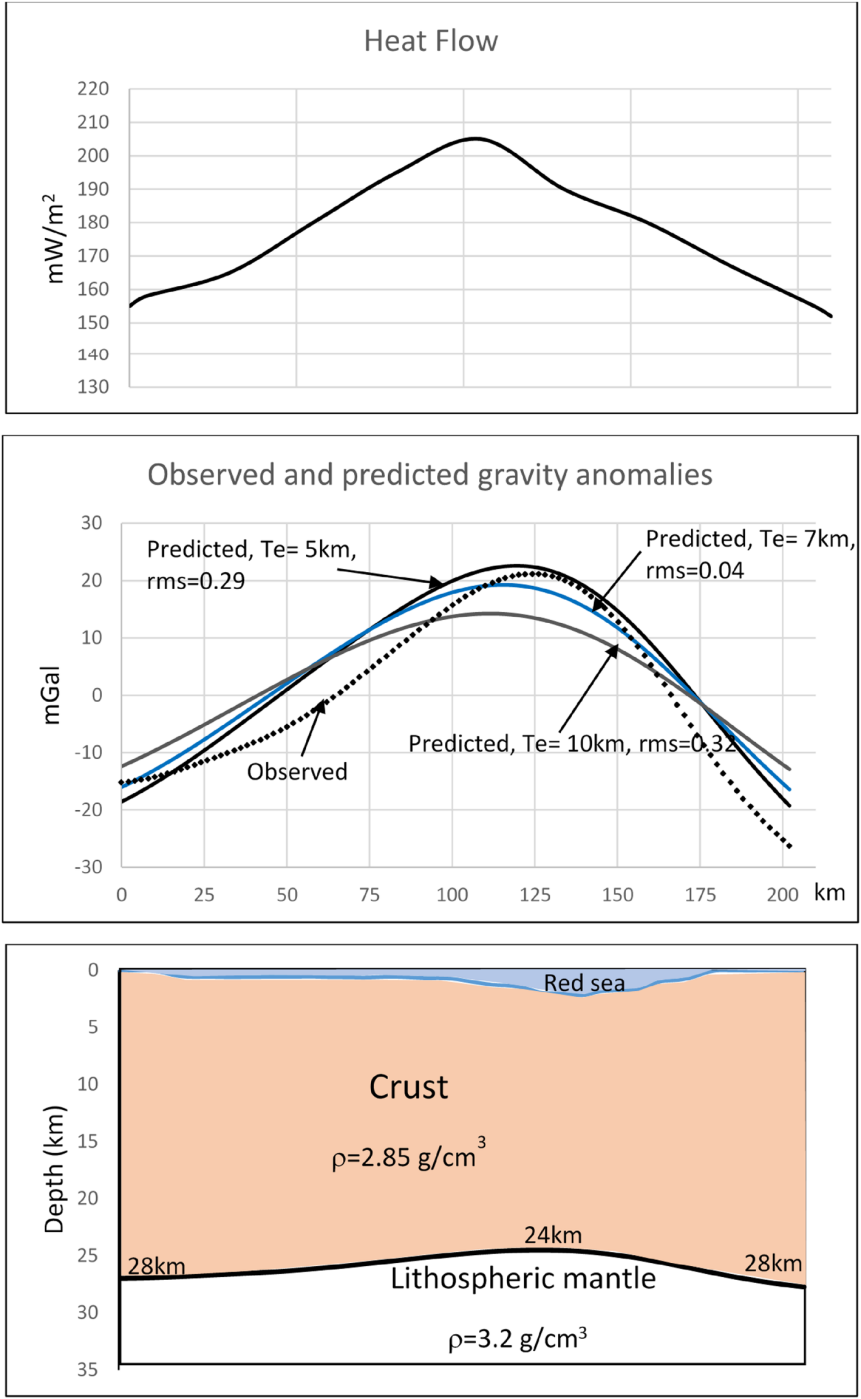
**(a)** The heat flow profile along the modeled profile (location shown in Fig. 1) derived from aeromagnetic anomalies analysis^9^. **(b)** The observed regional gravity anomaly profile and a set of predicted anomalies using a flexural model of lithosphere with different values of elastic thickness ***Te***. The residual RMS is shown for each calculated anomaly. The best fit is found for a flexural model of a lithosphere with elastic thickness ***Te*** = 7km. **(c)** The crustal model that explains the observed regional gravity anomaly, revealing a thinning of the crust from 28 km in coastal areas to 24 km beneath the axial rift of the Red Sea.

To find the best value of ***Te***, the misfit between observed and calculated data must be minimized. We used the iteration process using different values of ***Te***, until we obtained a calculated anomaly that matches the observed anomaly as well. The model with the lowest RMS value is considered to be the best fit. The lowest RMS is found for ***Te*** = 7km. The RMS increases for ***Te*** lower or higher than 7km. To better observe the comparison between observed and predicted anomalies, we present the observed anomaly profile superimposed on a set of predicted anomalies using different values of ***Te*** (Fig. 6b). Three predicted curves are shown in Fig. 6b: the curve obtained with ***Te*** = 7 km showing the lowest RMS (0.04), one curve obtained for ***Te*** lower than 7 km and one curve obtained with ***Te*** higher than 7 km.

The result of forward modeling shows that the better model that explains the observed anomaly is obtained by a flexural model with ***Te*** = 7 km. The flexural model suggests that there is a 4 km rise of the lithospheric mantle beneath the Mabahiss Deep (Fig. 6c), indicating that the anti-root is lower than what was predicted by the local isostatic model.

## Discussion

Understanding the mechanical properties of the Earth's outermost layer is made possible by calculating the elastic thickness of the lithosphere from gravity and bathymetry data. However, it is important to remember that the estimation of the lithospheric elastic thickness through gravity and bathymetry data encounters specific limitations and errors. Assumptions inherent in theoretical models, such as the uniformity of lithospheric properties or the absence of lateral variations, may introduce inaccuracies in the results. Furthermore, the calculated elastic thickness might be affected by inaccuracies in modeling approaches and uncertainties in gravity and bathymetry data. Still, the estimated value remains valuable to evaluate the flexural rigidity and the mechanical behavior of the lithosphere.

The elastic thickness (***Te***) of the continents has a broad range of values (5–110 km)^25^. Most of the continental areas show high values of ***Te*** ^26^. Furthermore, the values of ***Te*** may exhibit large spatial variations within the same plate^15,27^. The determined value of elastic thickness in NRS (***Te*** = 7 km) is notably low, indicating evidence of a weakened and stretched continental lithosphere beneath NRS. In the oceanic lithosphere, the thermal gradient has a major control on the rigidity and ***Te*** corresponds to a particular geotherm, whereas in continents, further factors may control the rigidity^27^. The weakness of the NRS crust can be attributed to various factors, including:

1. Temperature: based on measurements carried out in NRS, the heat flow is high and reaches 300 mW/m^2^ in the axial area of NRS^28^. Besides, the estimated Curie depth from aeromagnetic anomalies ranges between 6 and 9 km across the entire NRS region, and the heat flow varies between 160 and 240 mW/m^2^^1,9^. Figure 6a shows the estimated heat flow variation along a profile crossing the study area^9^. This high heat flow and the high temperature, which caused the rise of Curie depth points, weakens the lithosphere.
  Furthermore, a map delineating P-wave seismic velocity anomaly at a depth of 20 km is generated from seismic tomography inversion^29^. This map reveals a negative velocity anomaly along the NRS axis, suggesting the presence of relatively low-velocity material. The authors also constructed vertical W-E sections intersecting our study area, which display a negative P-wave velocity anomaly along the axial trough of the NRS within the crust and the mantle. This observation confirms the existence of high-temperature material along the axial trough of the NRS beneath the study area. Such relatively hot material could lead to the heating of the already thinned lithosphere, influencing its mechanical behavior and weakening it. Conversely, in the northernmost area of NRS, the seismic tomography inversion indicates a higher P-wave velocity^29^ and a deeper Curie point^1^. In this zone, the crust appears to remain unheated, suggesting behavior typical of a regular continental crust.
2. Tectonic activity: The continental lithosphere in the NRS is under extensional stress due to the northeastward movement of the Arabian plate relative to Eurasia that caused the rifting^30,31^. This extension induces a thinning of the lithosphere and a rising of the asthenosphere. This thinning is confirmed by the observed positive long-wavelength Bouguer anomaly along the NRS trough. As the lithosphere is extended, it will be weaker.
3. Faults and fractures: The existence of faults and fractures in the continental lithosphere can induce substantial weakening. The presence of active faults is evidenced by seismic activity across the entire NRS crust. The high frequency of seismicity observed in NRS with a dominance of low magnitude^1,2^ refers to the low ability of the lithosphere to deform elastically and its low ability to support the tectonic stress. This is confirmed by low cumulative seismic moment releases, which range between 12 and 15 N.m.^2,32^. These observations refer to a region characterized by high seismic strain and permanent deformation, suggesting a low ***Te*** and low ability to support the applied tectonic stresses without substantial deformation. However, active faults and fractures are constrained to shallow depths. Indeed, the hypocenters of earthquakes are limited to depths of 25 km in the NRS region, up to latitude 26°N. Conversely, they exceed 35 km beneath the northernmost regions of the Red Sea and the Gulf of Suez^1^. This observation implies that deformation becomes ductile below a depth of 25 km due to elevated temperatures. Such finding confirms the heating activity and the thinning of the lithosphere.

All these factors are associated with the tectonic extension and rifting that resulted in the formation of the Red Sea rift. As this area is under extension and is being rifted, the upper mantle uplift, and therefore the thermal gradient and the heat flow increase. The high thermal gradient is the main factor that induced the weakness of the crust beneath the NRS area. The low elastic thickness (***Te*** equal to 7km) of the study area fits most of continental elastic thickness estimates derived from extended areas. Indeed, the continental strength of active regions is relatively low, in the range of 0–20 km^26^. Also, using the wavelet method, the whole western part of the Arabian plate is characterized by low to moderate elastic thickness (10–30 km)^15^.

The weakened continental lithosphere in the NRS impacts its mechanical behavior. The rising of the asthenosphere^10,29^ and the development of rifting in the axis of the NRS are among factors that caused this weakness. There is controversy over the cause of rifting, whether it’s passive or active. While an active rifting process with a mantle flow for the whole Red Sea where the asthenosphere has an important role in its development is proposed^3^, other studies consider that the northern and central Red Sea regions are a passive rift caused by far-field stress that could be associated with subduction of the Arabian plate beneath the Zagros Mountains^33^. Based on a seismic reflection survey, the NRS is considered to be a passive non-volcanic rift^34^, whereas other authors, through analysis of geophysical data, consider that the NRS has reached the last stage of the rifting and could be in the first stage of the seafloor spreading^5,9,29^.

There is evidence of a lithospheric mantle uplift; however, the crust remains at least 24 km thick along the axial rift (Fig. 5c). The low elastic thickness (7 km) within such crustal thickness infers to a non-regular continental crust beneath the NRS region. The observed gravity anomaly is explained by a simple flexural model of an extended and weakened continental lithosphere.

The low elastic thickness is not indicative of an incipient oceanic crust. Given the absence of compelling evidence supporting the existence of oceanic crust material and oceanic spreading, we conclude that the NRS represents a continental crust that has been stretched and weakened. The attributed cause of this weakness is mainly heating activity, substantiated by heat flow measurements^28^ and heat flow estimated from aeromagnetic anomalies^1,9^ (Fig. 6a) . Due to the weakened state of the crust and the ongoing extension in the region, the NRS rift could evolve into a rupture, leading to the formation of oceanic crust.

## Conclusion

Using gravity and bathymetry data of the Mabahiss Deep in the Northern Red Sea, we derived the thickness variation of the crust and determined the elastic thickness (***Te***), which refers to the flexural rigidity of the lithosphere. The long-wavelength positive Bouguer gravity anomaly is attributed to crustal thinning and lithospheric mantle uplift that caused the apparition of the Red Sea. The crustal thickness varies from 28 km in the coastal area to 24 km along the axial rift of the NRS implying an isostatic anomaly. Using the forward modeling process, the best model that explains the gravity anomaly is a flexural model of a lithosphere with an elastic thickness ***Te*** equal to 7km. This value is considered low and infers a non-regular continental crust; however, it’s not indicative of an incipient oceanic crust as suggested by some studies. Given the lack of compelling evidence supporting the existence of oceanic crust material and oceanic spreading, we conclude that the NRS represents a continental crust that has been stretched and weakened. Various factors contribute to the weakness of the lithosphere, including temperature, tectonic extension, and the presence of active faults. However, the primary cause is the heating activity, evidenced by the high heat flow. Due to the weakened state of the crust and the ongoing extension in the region, the Northern Red Sea rift could potentially evolve into a rupture, leading to the formation of oceanic crust.

## Data Availability

Gravity and bathymetry data used in this study are available and can be downloaded from the website of Bureau Gravimetrque International: https://bgi.obs-mip.fr/data-products/grids-and-models/wgm2012-global-model.
